# Inhibitory effects of cold atmospheric plasma on population growth of the carob moth, *Ectomyelois ceratoniae* (Lepidoptera: Pyralidae) in laboratory

**DOI:** 10.1371/journal.pone.0294852

**Published:** 2023-11-27

**Authors:** Mahmoud Soufbaf, Mojtaba Nohekhan, Mahdieh Bakhtiari

**Affiliations:** 1 Agriculture Research School, Nuclear Science and Technology Research Institute (NSTRI), Karaj, Iran; 2 Plasma and Fusion Research School, Nuclear Science and Technology Research Institute (NSTRI), Tehran, Iran; Harran Universitesi, TURKEY

## Abstract

Potential sterilizing effects of the atmospheric cold plasma on the carob moth, *Ectomyelois ceratoniae* Zeller (Lepidoptera: Pyralidae) was studied under laboratory conditions by means of life history experimentation. The results showed that the population growth parameters of the carob moth decreased in all periods of 15 to 60 sec of plasma treatments applied on 1 –day–old eggs. Overall, 19.5% and 23.8% of reproduced eggs were fertile when the experimental males and females mated with normal moths in indirect treatments of 15 and 30 sec, respectively. The highest intrinsic rate of population increase, *r*, was in control (0.11 day^-1^) and the highest decrease in this parameter was in the 30 sec direct treatment (- 0.073 day^-1^). The results showed that cold plasma had an acceptable potential to sterilize the pest if plasma was applied at egg stage. This potential will be explored from the perspective of insect sterility technique and the genetical / physiological mechanisms involved should be studied in future.

## Introduction

The carob moth, *Ectomyelois ceratoniae* Zeller (Lepidoptera: Pyralidae) is the key pest of pomegranates, date, figs and some stored crops such as pistachios [1]. Depending on variety of either biotic or abiotic circumstances, the damage caused by this pest varies in different years. Currently, this pest is active in almost all pomegranate orchards and it destroys ⁓30 to 50% of the pomegranates annually in Iran. Due to hidden feeding activity of larvae inside fruits, chemicals’ spray is not effective and therefore, scientific studies on the control of the pest have been directed to alternative methods [2, 3]. The carob moth overwintering takes place in the form of larvae of different instars inside fruits usually that have fallen under the trees or remained on the tree or on/in the stored products. After completing the physiological time, in early spring, leading moths start to emerge gradually and soon after to mate and reproduce [4].

Plasma is an ionized gas that consists of active chemical species with antimicrobial properties including electrons, ions and neutral molecules and atoms, depending on the temperature at which it is produced in two cold and hot types. The gas used in the plasma production systems can be air, noble gases (helium and argon), nitrogen, carbon dioxide, oxygen or a combination of different gases and the formation of ionization products in the plasma process depends on the type of gas used, voltage, current rate and frequency, relative humidity, process time and device power [5, 6]. The chemical active species overcome the defense system of various microorganisms by creating a deadly environment for them via injuring the genome, proteins, lipids and cell membrane leading to their death [7, 8].

Effects of the cold plasma on insects and mites were first studied by [9] on *Sitophilus granarius* L. (Coleoptera: Curculionidae), then by [10] on cigarette beetle *Lasioderma serricorne* Fabricius (Coleoptera: Ptinidae) and then by [11] on western flower thrips *Frankliniella occidentalis* f. *brunnescens* Priesner (Thysanoptera: Thripidae), tobacco thrips *Thrips tabaci* Lindeman (Thysanoptera: Thripidae), a species of Aedes mosquito, the cockroach *Blatella germanica* L. (Dictyoptera: Blatellidae) and the mite *Tetranychus urticae* Koch (Acari: Tetranichidae). Moreover, [12] studied the use of plasma jet against *Ephestia kuehniella* Zeller (Lepidoptera: Pyralidae). In any case, there are only a handful of studies on the use of plasma–based methods to knock down various pests, but due to the unique ability of this method in the management of especially insect pests, this procedure is developing considerably in this field.

Application of plasma against different life stages of various insect pests as a killing strategy is tested by many workers as referenced already in part in current paper. However, after some initial tests, we found that plasma application in time periods less than 120 sec had no killing effect on either immatures (egg, larva, and pupa) or adults of the carob moth and some effects such as prolonging the developmental time was observed totally. In other words, plasma–generated chemical species have a superficial penetration depth and all reactions (physical and/or chemical) are on the surface of the subjects [13]. Due to this feature and following the work done by [10] on cigarette beetle *L*. *serricorne* in which they showed nearly 70% sterility of the beetles treated by atmospheric plasma for 45 sec just after adults’ emergence, we concluded that plasma species that have high chemical variation might have a sterilizing effect on the 1 –day–old egg stage of the carob moth because treatment of newly deposited eggs could be the most similar to treatment of newly emerged adults (that showed 70% sterility in the last work) in terms of reproduction physiology. This stage also is more suitable candidate to be treated in plasma chamber as is not mobile like adults of the moth. Diameter of a given egg of the carob moth is about 1 mm and the penetration depth of atmospheric plasma inside the eggs especially through the micropyle could guarantee some chemical and/or physical reaction of the primary cytoplasmic cells with plasma species. Of course, imaginal discs in epidermal cells of lepidopteran larvae are other candidate that can be affected by superficial plasma reactions because they are in a narrow depth beneath the larval epidermis; whereas, mobility of larvae and adults inside the plasma chamber caused un–uniformity in treatment of plasma on these stages. In total, due to observed nonlethal effects and various reported effects of cold plasma on different insect species e.g. the sterility of the cigarette beetle mentioned above, we studied the potential sterilizing effect of cold atmospheric plasma on 1 –day–old eggs of the carob moth via a life history experimentation.

## Materials and methods

### Insect rearing

In order to rearing the carob moth under constant environmental conditions, infested pomegranates were collected from pomegranate orchards of Lorestan province by the end of October and early November. These fruits were kept in a small isolated area in clean rooms with constant environmental conditions of 29 ± 1°C, 75 ± 5% RH and photoperiod 16/8 (L/D). After emergence of the adults, mass rearing was stablished on an. artificial diet consisting of brewer’s yeast (Blue Life, Germany) (23 g), glycerin (Dr.MOJALLALI industrial chemical complex Co., Iran) (150 ml), wheat bran (600 g), sucrose (120 g), vitamin C (Qulian France packaged by Suja–Pars company, Iran) (6 g), aeromycin (Livestock medicines production of Iran) (6.7 g), lysine (L-Lysin. PT. Cheiljedang, Indonesia) (3 g) and distilled water (250 ml) [14] and continued until the end of experiments under above mentioned environmental conditions. Adults emerged in the flight cages (70 × 70 × 100 cm) were collected by an electric aspirator and were transferred to the egg laying cylinders (15 cm diameter × 25 cm height). Eggs were collected daily until the death of the majority of the population in each cylinder and the egg loaded sheets were deposited on the artificial diet’s trays.

### Plasma treatment

**Fig 1:**
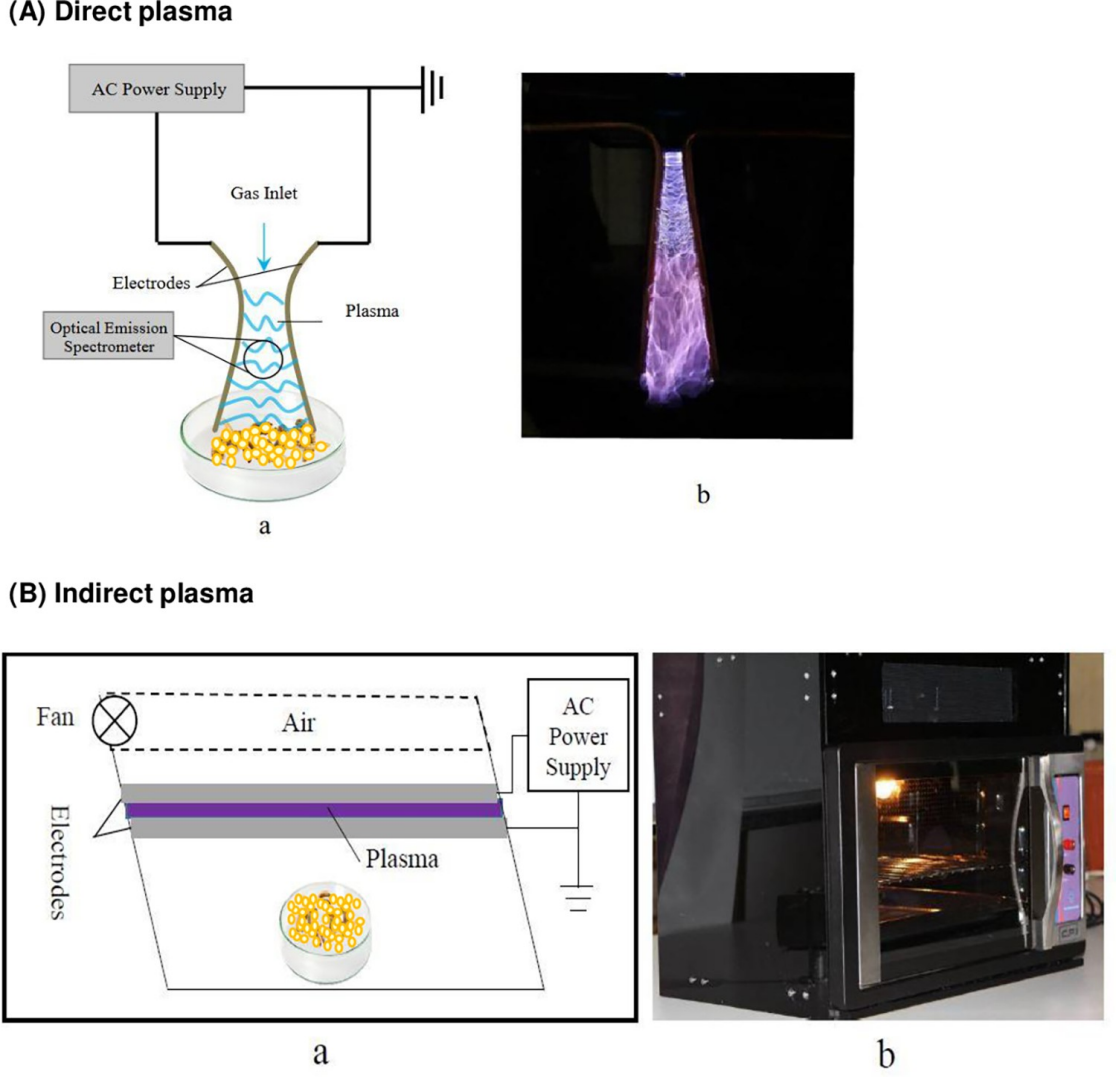
Atmospheric cold plasma set up for direct (A) and indirect exposure (B) of the eggs of *Ectomyelois ceratoniae*. a and b show schematic and real plasma environments used in this research, respectively.

The atmospheric cold plasma based on gliding arc discharge (GAD) is formed when a high electric potential is applied between two metal electrodes, without any insulation. Strong sparks and arc discharge are formed by applying this voltage. The flow of a gas, which is often air, expands on the electric discharge zone after the sparks are formed, and prevents the electric discharge zone from overheating. In this experiment, a power supply with variable voltage 0 – 15kV and a sine frequency of 50 kHz was used to produce the discharge (Fig 1). The 1 –day–old eggs were treated through two different plasma environments; first was direct exposure to sparks as shown in the Fig 1(A), and second was to treat eggs with no direct exposure to sparks in an environment in which only plasma induced chemical species were active (Fig 1B).

### Biological and population growth parameters

In order to investigate the effect of different plasma treatments (15, 30 and 60 sec, both forms of direct and indirect exposure) on the development period and the survival of the immature stages of the carob moth, a cohort of 40 1 –day–old eggs was treated for each treatment and then the treated eggs were transferred individually into 9 cm diameter Petri dishes containing artificial diet (about 3 g of diet in each Petri dish). The Petri dishes were checked and the food of the larvae was replaced daily with fresh one. Recording data of all three treatments in two cases of direct and indirect exposure to plasma was done simultaneously based on a completely randomized design in insectary with the above–mentioned environmental conditions. In total, there was 320 Petri dishes that were censused daily in the experiment. The parameters related to the immature stages such as the incubation period, larval, pupal and developmental time and the survivorship were recorded and this investigation continued until the emergence of adult insects. After the emergence of adults from plasma treated eggs, every pair was transferred to small cylindrical cages (12cm h × 6cm d) for mating and laying eggs till the death of the last female. In case of male death, a male was added to the cage. In order to study the role of either plasma treated female or plasma treated male separately in population growth of the moth, they were mated individually with untreated males and females, respectively. In fact, there were three cohorts of the moths identified treated female + treated male, treated female + untreated male, and untreated female + treated male and only the first cohort (treated female + treated male) was studied by the two sexes technique. The number of laid eggs and the lifespan of male and female insects were checked and recorded daily. These surveys were continued until the death of all insects and the recorded data were used to form a life table. The experiments were conducted in the insectary of Karaj Agricultural Research School with a temperature of 29 ± 1°C, 70 ± 5% RH and a photoperiod of 14/10 h (L/D). Population growth parameters were calculated using the data obtained from the demography of the tested insects via the life table theory of two sexes [15]. Age–stage specific survival rate (*s*_*xj*_), age–specific fecundity (*m*_*x*_), age–stage specific life expectancy (*e*_*xj*_), age–stage specific reproductive value (*v*_*xj*_), and the main life table parameters including net reproductive rate (*R*_*0*_), intrinsic rate of population increase (*r*), finite rate of population increase (*λ*), mean generation time (*T*) and the gross reproduction rate (GRR) were calculated according to formulae presented in Table 1. The age–stage–specific survival rate indicates the probability of the egg surviving to age x while in stage j. In addition to a detailed description of survival, this parameter describes the transition from one age to the next one. Age–stage–specific fecundity (*f*_*xj*_) shows the number of offspring produced by each individual at age x and stage j. The age–stage specific value of reproduction is the expectation of individuals at age x and stage j to reproduce the next offspring [15].

**Table 1:** Life history parameters’ notations, formulae and units used in TWOSEX–MS Chart software [15].

Parameter	Notation	Formula	Unit
The intrinsic rate of increase	*r*	$\sum_{x=0}^{\beta }e^{-r(x+1)}L_{x}m_{x}=1,(\beta =the\;last\;day\;of\;the\;last\;female^{\prime }\;age)$	Day^-1^
The finite rate of increase	Lambda, *λ*	*λ* = e^r^	Day^-1^
The net reproductive rate	*R*_*0*_	$R_{0}=\sum_{x=1}^{\omega }\sum_{j=1}^{m}s_{\mathrm{xj}}f_{xj},(\omega =the\;days\;of\;life\;span,\;m=the\;developmental\;stage)$	Offspring/individual
The mean generation time	*T*	$T=\frac{LnR_{0}}{r}$	Day
The gross reproduction rate	GRR	$GRR=\sum_{x=\alpha }^{\beta }m_{x},(\alpha ,\;and\;\beta \;are\;the\;first\;and\;the\;last\;day\;of\;oviposition,\;resp\;repectively)$	Offspring

### Statistical analysis

The data were analyzed using TWOSEX–MS Chart software [15]. Bootstrap method with 100,000 repetitions was used to replicate the parameters of the life table. Also, the statistical difference between biological characteristics and population growth parameters was compared using paired–bootstrap test (P< 0.05). A completely randomized design (CRD) was used to evaluate the effect of plasma treatments on the life history of the pest. To compare the statistical difference of the percentage of reproduced fertile eggs in the treated female + untreated male, and untreated female + treated male treatments one-way ANOVA was used and the mean difference between the treatments was analyzed using the Holm–Sidak test at the 5% probability level. Before analysis, except for the life table parameters, all data were examined for normality using Shapiro–Wilk test (P< 0.05). Normality of life table parameters is assured due to large number of bootstrap (B = 100000) [15]. All statistical tests and plots were performed using SigmaPlot 12.3.

## Results and discussion

**Fig 2:**
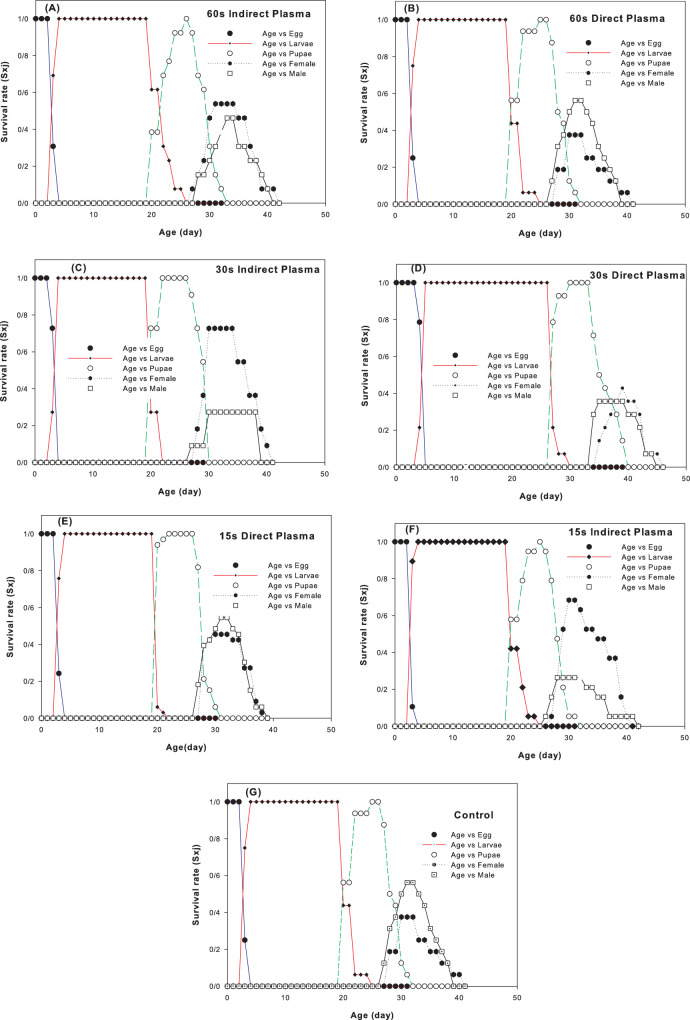
The age–stage survival rate (*s*_*xj*_) of *Ectomyelois ceratoniae* under different plasma treatments.

**Fig 3:**
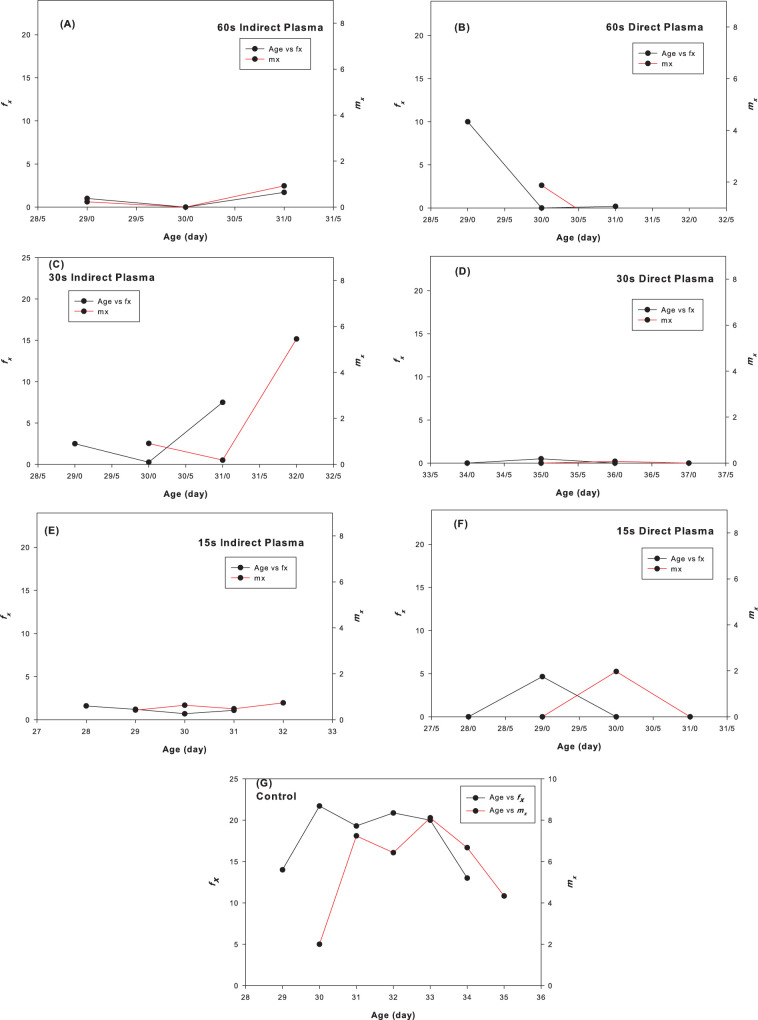
Age–specific fecundity (*m*_*x*_) and female fecundity (*f*_*x*_) of *Ectomyelois ceratoniae* under different plasma treatments.

**Fig 4:**
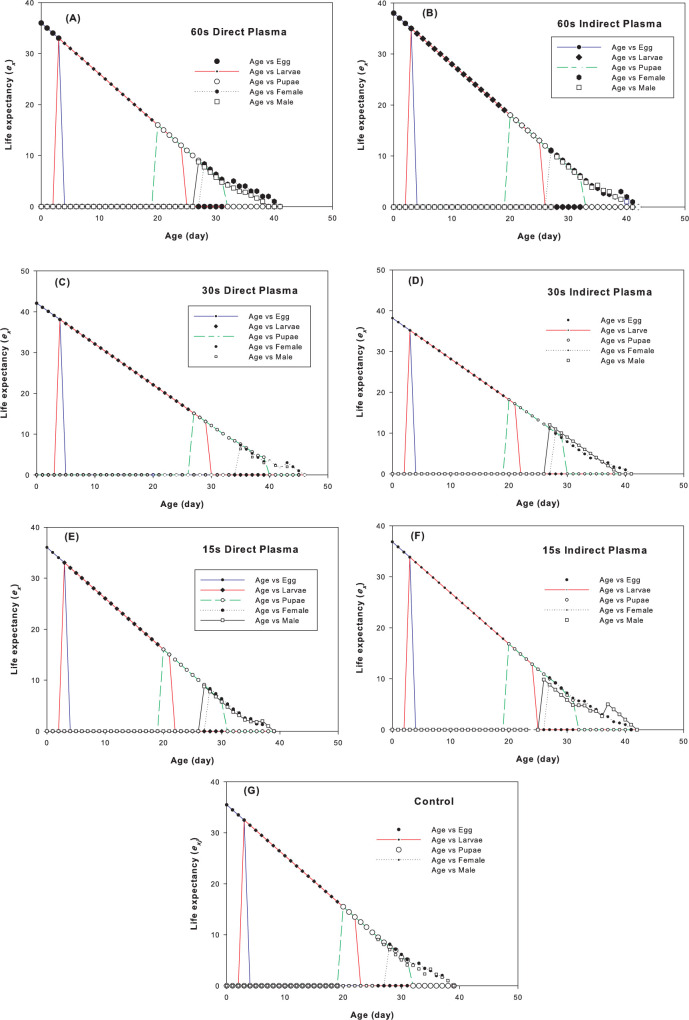
Age–stage life expectancy (*e*_*xj*_) of *Ectomyelois ceratoniae* under different plasma treatments.

**Fig 5:**
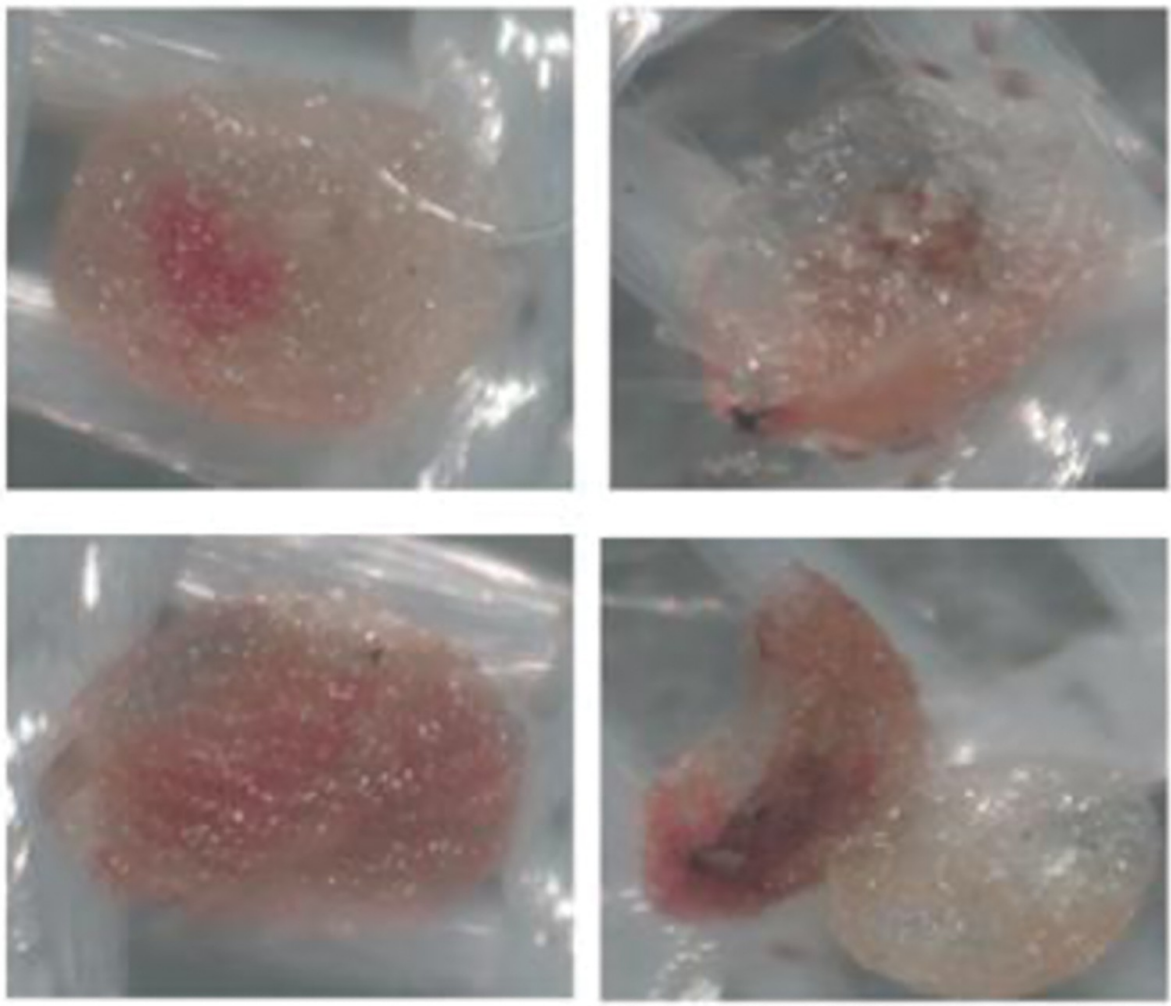
Dead embryos observed inside the eggs of *Ectomyelois ceratoniae* in a period of about 180 sec of the cold atmospheric plasma.

The age–stage survival rate (*s*_*xj*_) of the carob moth under different plasma treatments is shown in Fig 2. Different plasma treatments did not affect the survival patterns of all developmental stages. However, survival of the larva under 30 sec direct plasma was more lasting than the respective correlate in the other treatments and either larval deaths or transformations to the pupal stage was occurred about 7 days later accordingly. The rate of mortality of adult males as the slope of their survival pattern was the slowest in 15 sec indirect plasma while such correlate for females was occurred in 60 sec direct plasma (Fig 2). Age–specific fecundity (*m*_*x*_) and female fecundity (*f*_*x*_) of the carob moths under different plasma treatments (Fig 3) show a decrease in *m*_*x*_ and *f*_*x*_ under different plasma treatments compared to the control. These changes include not only the size of fecundity, but also the pattern of temporal changes of fecundity. The oviposition window decreased 2 times in 15 sec indirect plasma and 3 times in other treatments compared to control (Fig 3). This parameter is related properly to the population increase ability of the moths in terms of their time opportunity to increase their population size. Plasma treatment did not affect the pattern of the decreasing age–stage life expectancy (*e*_*xj*_) of the carob moth (Fig 4) which indicates that plasma has no significant effect on the life span of the pest. The initial results showed that in treatments lower than 120 sec, the carob moth passes a successful immature period and the eggs hatch normally, but with the increased exposure time, especially in times of about 3 min, most of the embryos inside the eggs suffer from growth defects and death (Fig 5). However, the egg hatchability in all the experimental treatments was 100% and except for reduced fertility of the pest in either treated female + untreated male or treated male + untreated female treatments that was remained unaffected among all plasma treatments, life history parameters that were calculated in treated female + treated male showed a slight difference among different plasma treatments.

The results showed a significant and multiple fold decrease in the percentage of fertile eggs obtained in different plasma treatments compared to the control (*F*_6,30_ = 200.4, P < 0.001). The highest percentage of fertilized eggs was observed in the control treatment (98.44 ± 2.17) and the lowest was in the direct 30 sec treatment (0.00 ± 0.00). The results of the means’ comparison showed a significant difference between the control and all the plasma treatments in the percentage of fertilized eggs and no difference between the other treatments (Table 2).

**Table 2:** Mean (±SE) comparison of the percentage of fertile egg reproduced from mating of the plasma treated male of *Ectomyelois ceratoniae* with untreated female.

Plasma treatment (duration and type)	Mean ± SE
**30 s, indirect**	8.23 ± 7.44^b^ (n = 3)
**30 s, direct**	0.00 ± 0.00^b^ (n = 5)
**control**	98.44 ± 0.97^a^ (n = 5)
**15 s, direct**	2.51 ± 1.88^b^ (n = 9)
**15 s, indirect**	4.09 ± 1.83^b^ (n = 5)
**60 s, indirect**	2.78 ± 2.78^b^ (n = 4)
**60 s, direct**	2.93 ± 2.75^b^ (n = 6)

Data with different letters in the same column are significantly different at p < 0.05 by Holm–Sidak test

Similarly, in treated female + untreated male treatment potential effects due to plasma treatments are come with males that are not applicable in cohort–based life history experimentation. Therefore, e analysis of fertility was done by means of a one–way analysis of variance. The results showed a significant and multiple fold decrease in the percentage of fertile eggs obtained in different plasma treatments compared to the control (*F*_6,50_ = 238.63, P < 0.001). The highest percentage of fertilized eggs was observed in the control treatment as expected (99.28 ± 1.25) and the lowest in the direct 30 sec treatment (0.00 ± 0.00). Similar to the results related to males, the average comparison results showed a difference between the control and all the plasma treatments in the percentage of fertile eggs and no difference between the other treatments (Table 3). The results of the bootstrap analysis of the life table parameters of the carob moth under different plasma treatments showed a decrease in the intrinsic rate of pest population increase (Table 4). The highest and the lowest intrinsic rate of population increase (*r*) were related to the control and the treatment of 30 sec of direct plasma, respectively. In this treatment, the population growth rate was negative. Similarly, the net reproductive rate (*R*_*0*_) was the highest in the control treatment and the lowest in the 30 s direct treatment (Table 4). Also, the finite rate of population increase (*λ*) showed the highest value in the control and decreased by 1.19 times in the direct 30 sec treatment compared to the control. The direct 30 sec treatment led to an increase in the mean generation time in the carob moth for approximately 6 days compared to other plasma treatments and an increase of approximately 4 days compared to the control (Table 4). The gross reproductive rate, GRR was significantly reduced compared to the control in accordance with other reproductive parameters (Table 4).

**Table 3:** Means (± SE) of the percentage of fertile egg reproduced from mating of the plasma treated female of *Ectomyelois ceratoniae* with untreated male.

Plasma treatment (duration and type)	Mean ± SE
**30 s, indirect**	3.30 ± 1. 026^b^ (n = 5)
**30 s, direct**	0.00 ± 0.00^b^ (n = 6)
**control**	99.28 ± 0. 511^a^ (n = 6)
**15 s, direct**	4.37 ± 1.41^b^ (n = 13)
**15 s, indirect**	5.19 ± 2.21^b^ (n = 13)
**60 s, indirect**	2.80 ± 1.60^b^ (n = 8)
**60 s, direct**	4.17 ± 3.97^b^ (n = 6)

Data with different letters in the same column are significantly different at p < 0.05 by Holm–Sidak test

**Table 4:** Means (± SE) of life history parameters of *Ectomyelois ceratoniae* under different plasma treatments.

Plasma treatment (No of emerged adults of two sexes)	Life history parameters (mean ± SE)				
	GRR	*T*	*λ*	*R*_*0*_	*r*
**15 sec, direct (15♀+18♂)**	1.97 ± 0.95^b^	30.00 ± 0.82^d^	1.02 ± 0.0181^b^	1.97 ± 0.95^b^	0.0226 ± 0.0178^b^
**15 sec, indirect (13♀+6♂)**	2.26 ± 0.88^b^	30.66 ± 1.13^cd^	1.03 ± 0.0148^b^	2.26 ± 0.88^b^	0.03 ± 0.0145^b^
**30 sec, direct (7♀+7♂)**	0.07 ± 0.05^c^	36.00 ± 7.15E-7^a^	0.93 ± 0.0112^c^	0.0714 ± 0.0546^c^	-0.0733 ± 0.0001^c^
**30 sec, indirect (8♀+3♂)**	6.54 ± 4.92^b^	31.68 ± 0.66^bc^	1.06 ± 0.0342^ab^	6.54 ± 4.9^bc^	0.06 ± 0.0331^ab^
**60 sec, direct (6♀+10♂)**	1.94 ± 1.78^bc^	30.06 ± 0.87^cd^	1.02 ± 0.0493^bc^	1.94 ± 1.78^bc^	0.022 ± 0.0502^bc^
**60 sec, indirect (7♀+6♂)**	1.15 ± 0.59^b^	31.60 ± 2.20^abcd^	1.00 ± 0.0185^b^	1.15 ± 0.59^b^	0.00453 ± 0.0186^b^
**Control (7♀+14♂)**	34.87 ± 12.23^a^	32.37 ± 0.42^b^	1.11 ± 0.0142^a^	30.48 ± 10.97^a^	0.11 ± 0.0129^a^

Data with different letters in the same column are significantly different at p < 0.05 by Holm–Sidak test

Ignoring statistical significancy, zero offspring in 30 sec plasma in either direct or indirect exposure that was done in completely different set–ups is the main controversial but important part of the current work. Higher fertile eggs reproduced in 60 sec treatments, however, could be related to some heterogeneities in distribution of eggs in Petri dishes inside plasma chambers and more importantly, to random movements and subsequently random collisions between chemical species and eggs with diverse energies. Therefore, to be more consistent in getting a logical trade–off we need spectral chemical analysis of produced plasma in each chamber in each run to find associations between chemical elements composition of the plasma material and the occurrence of any physical or biological event there.

**Fig 6:**
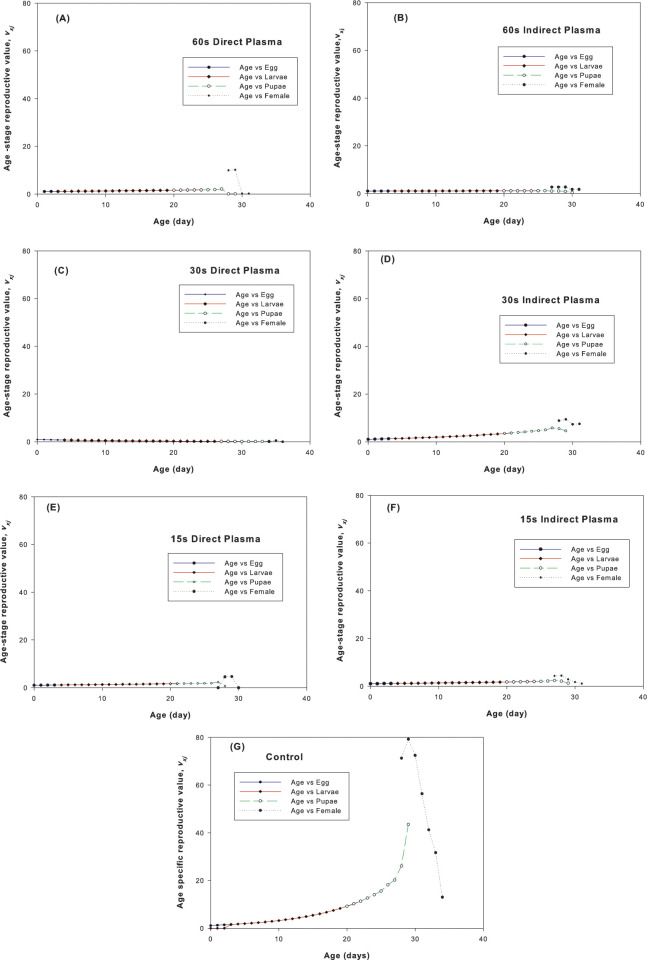
Age–stage reproductive value (*v*_*xj*_) of *Ectomyelois ceratoniae* under different plasma treatments.

The age–specific reproduction value of the carob moth under different plasma treatments in this research is shown in Fig 6. The results showed that the maximum fertility in all treatments was similar and around days 29 to 30^th^, and only in the direct 30 sec treatment, this peak was weak and transferred to the days after 30^th^ days. Also, the highest rate of oviposition was in the control and in the first days after the appearance of female insects, which decreased with age and finally reached to zero. In addition, among the various biological stages, the pupal period had a higher reproductive value than the other stages.

There is a tone of works on the life history experimentation of high variety of insect pests on a wide spectrum of host plants and artificial / semi artificial diets considering different controlling treatments. However, focusing the topic of application of plasma against insect pests, obviously, except for mortality percentage, there is no study reporting life table parameters of any because the only purpose of that works has been ’knockdown’ so far. For instance, [16] in studying the effect of plasma jet with argon gas and argon/oxygen and argon/nitrogen combinations showed that this method has a high potential in controlling warehouse pests such as *Tribolium castaneum* Herbst (Coleoptera: Tenebrionidae) and *Tribolium confusum* Jacqueline du Val. (Coleoptera: Tenebrionidae). In a recent study, the percentage of experimental insect mortality in different exposure time treatments and gas concentration increased greatly. [17] in the study of the biological response of several pest insects including *Aphis gossypii* Glover (Hemiptera: Aphididae), *Bemisia tabaci* Gennadius (Hemiptera: Aleyrodidae), *Helicoverpa armigera* Hubner (Lepidoptera: Noctuidae), *Thrips palmi* Karny (Thysanoptera: Thripidae) and a species of mite *Tetranychus kanzawai* Kishida (Acari: Tetranychidae) to oxygen species released during the plasma jet process showed that *B*. *tabaci* shows the fastest knockdown in 38 sec when it is exposed to plasma for less than 3 min. The results of these researchers showed 100% effectiveness of this technique against all 5 species studied. [18] showed that 100% mortality was achieved in the experimental population of *T*. *confusum* and *E*. *kuehniella* with plasma jet treatment of 20 sec. In a recent study, [19], reported an approximate duration of 14 min of atmospheric/argon cold plasma for complete destruction of *Plodia interpunctella* (Hübner) (Lepidoptera: Pyralidae) on pistachios. In another new study, [20] using two gases, argon and helium, showed that helium gas was more successful in controlling *T*. *castaneum* than argon gas and the best time to achieve the maximum mortality was 90 sec. In the later study, the most resistant biological stages of the pest were reported in the larva, pupa and adult beetles. [21] showed the ability of cold plasma to destroy the larvae and pupae of *T*. *castaneum* and noted that reactive oxygen species in the environment caused the oxidation of fat in the cell membrane of living insects. Also, in the last research, the reduction of lipase enzyme activity due to the effect of plasma was proven in *T*. *castaneum*. The current results showed a decrease in fertility and a decrease in the pest population growth rate due to cold plasma effects. In a similar and unique effort done by [10], up to 75% sterility was reported after 45 sec exposure of *L*. *serricorne* newly adults to atmospheric cold plasma. These results can strengthen the possibility of sterilization of the carob moth with this method, although the mechanism of this event needs additional biological tests, which will be addressed in the next step of the research.

## Conclusions

Newly deposited eggs were exposed to plasma generated chemical species and after completing life span, the adult moths showed more than 95% sterility after mating with untreated counterparts. There are many chemical species that are reproduced through plasma production such as free radicals, various ions, H_2_O_2_ etc., however high energy electrons could be a good candidate for sterilization of the 1 –day–old eggs of the carob moth. These electrons could affect potentially some cells, namely Pole cells [22], who will become the gametes of the treated adults. However, embryonic cell clusters namely imaginal discs are other candidate whose damage could lead to reproductive process setback. We recommend to do complete genetical analyses to track the potential events aiming to prove sterilization occurrence due to cold plasma mechanistically. Also, we suggest that less than 60 sec of cold atmospheric plasma against different immature stages of the carob moth had no killing effects and so is not applicable as disinfection technique before warehouse of host crops of the carob moth. In such efforts to eradicate the pest from crops before storing in warehouses, if penetration of plasma born chemical species is not the problem, applying either high voltage plasma in very short pulses in alternation in warehouses that are emptied deliberately to disinfestation or treating crops in thin layers of mobile rows is certainly effective and more beneficial than using synthetic chemicals like fumigants. In atmospheric plasma application, just cost of electricity usage and equipment trivial break-down must be considered while in using chemicals, potential side effects as critical harmful residues are sufficient to be considered. However, following our focus in the current study, replacing atmospheric plasma to irradiation (either gamma or X) in the process of Sterile Insect Technique could be undoubtedly an evolutionary improvement in terms of both costs and personnel’ immunity considerations.

## Supporting information

S1 Text Plasma 30gm. (TXT)

S2 Text Plasma 30m. (TXT)

S3 Text Plasma 60gm. (TXT)

S4 Text Plasma 60m. (TXT)

S5 Text Plasma 15gm. (TXT)

S6 Text Plasma 15m. (TXT)

S7 Text Plasma control. (TXT)
